# The long-term prognostic and predictive capacity of cyclin D1 gene amplification in 2305 breast tumours

**DOI:** 10.1186/s13058-019-1121-4

**Published:** 2019-02-28

**Authors:** Arian Lundberg, Linda S. Lindström, Jingmei Li, J. Chuck Harrell, Eva Darai-Ramqvist, Emmanouil G. Sifakis, Theodoros Foukakis, Charles M. Perou, Kamila Czene, Jonas Bergh, Nicholas P. Tobin

**Affiliations:** 1 Department of Oncology and Pathology, Karolinska Institutet and University Hospital, Stockholm, Sweden; 2 Department of Biosciences and Nutrition, Karolinska Institutet and University Hospital, Stockholm, Sweden; 3 Department of Medical Epidemiology and Biostatistics, Karolinska Institutet, Stockholm, Sweden; 4 Human Genetics, Genome Institute of Singapore, Singapore, Singapore; 5 Department of Pathology, Virginia Commonwealth University, Richmond, VA USA; 6 Department of Pathology and Cytology, Karolinska Institutet and University Hospital, Stockholm, Sweden; 7 Department of Genetics, The University of North Carolina at Chapel Hill, Chapel Hill, NC USA; 8 Department of Public Health, Oxford University, Oxford, UK

**Keywords:** CCND1 gene amplification, Gene expression, Breast cancer, PAM50, Luminal A, CDK4/6, BCSS

## Abstract

**Background:**

Use of cyclin D1 (*CCND1*) gene amplification as a breast cancer biomarker has been hampered by conflicting assessments of the relationship between cyclin D1 protein levels and patient survival. Here, we aimed to clarify its prognostic and treatment predictive potential through comprehensive long-term survival analyses.

**Methods:**

*CCND1* amplification was assessed using SNP arrays from two cohorts of 1965 and 340 patients with matching gene expression array and clinical follow-up data of over 15 years. Kaplan-Meier and multivariable Cox regression analyses were used to determine survival differences between *CCND1* amplified vs. non-amplified tumours in clinically relevant patient sets, within PAM50 subtypes and within treatment-specific subgroups. Boxplots and differential gene expression analyses were performed to assess differences between amplified vs. non-amplified tumours within PAM50 subtypes.

**Results:**

When combining both cohorts, worse survival was found for patients with *CCND1*-amplified tumours in luminal A (HR = 1.68; 95% CI, 1.15–2.46), luminal B (1.37; 1.01–1.86) and ER+/LN−/HER2− (1.66; 1.14–2.41) subgroups. In gene expression analysis, *CCND1*-amplified luminal A tumours showed increased proliferation (*P* < 0.001) and decreased progesterone (*P* = 0.002) levels along with a large overlap in differentially expressed genes when comparing luminal A and B-amplified vs. non-amplified tumours.

**Conclusions:**

Our results indicate that *CCND1* amplification is associated with worse 15-year survival in ER+/LN−/HER2−, luminal A and luminal B patients. Moreover, luminal A *CCND1*-amplified tumours display gene expression changes consistent with a more aggressive phenotype. These novel findings highlight the potential of *CCND1* to identify patients that could benefit from long-term treatment strategies.

**Electronic supplementary material:**

The online version of this article (10.1186/s13058-019-1121-4) contains supplementary material, which is available to authorized users.

## Background

Mammalian cyclin D1, first identified in 1991 [1, 2], mediates G1 to S-phase transition in the cell cycle along with its binding partners CDK4/6. Overexpression of its protein has been found in 50–70% of breast cancers [3–6] whilst amplification of its corresponding gene, *CCND1*, has been shown in approximately 9–30% of cases [7–11]. These figures indicate that processes other than gene amplification are also responsible for overexpression of the protein.

The *CCND1* gene maps to the 11q13 breast cancer risk locus and the majority of breast tumours bearing amplification of the gene are oestrogen receptor (ER) positive [12–15], are of luminal B subtype [12, 13], and overexpress cyclin D1 protein [9, 12, 14, 15]. Most notably, patients with ER-positive *CCND1*-amplified tumours also show reduced survival times [9, 10, 12, 15]. The use of *CCND1* amplification as a biomarker in a clinical setting has been hampered by conflicting assessments of the relationship between cyclin D1 protein levels and clinico-pathological parameters. To be explicit, overexpression of cyclin D1 protein has been linked to both better [9, 16–18] and worse prognosis [19, 20] in breast cancer patients. These results are in clear contrast to the consistent prognostic message of tumour aggressively and reduced survival provided by *CCND1* amplification.

Among current research needs in breast cancer, biomarkers capable of helping to predict late recurrences are urgently needed [21]. In this regard, molecular biomarkers and in particular gene expression signatures may provide some utility [22–24]. Here, we aimed to determine if amplification of *CCND1* can function as a molecular biomarker for long-term breast cancer survival and more generally to comprehensively characterise its prognostic and treatment predictive capacity. In order to achieve this, we performed an integrative analysis combining gene amplification, gene expression and clinico-pathological data in two large cohorts of 1965 and 340 patients, respectively, with over 15 years of follow-up. We focus on clinically relevant patient subgroups including all, ER-positive/lymph node negative/human epidermal growth factor receptor 2 negative (ER+/LN−/HER2−), ER+/LN+/HER2−, HER2+ and triple negative breast cancers (TNBCs), as well as the PAM50 subtypes (luminal A, luminal B, HER2-enriched and basal-like) and treatment-specific subgroups (patients who received chemotherapy, endocrine therapy, both sequentially or untreated).

## Materials and methods

### Study population and specimens

Cohort 1 is comprised of tumours from the METABRIC study, and patient/tumour characteristics, treatments received and clinical endpoints have been previously described in detail [25, 26]. Briefly, this cohort consists of a total of 1992 primary breast cancers from patients in the UK and Canada with a median follow-up accounting for censoring of 10.2 years. Of the original 1992 patients, 1965 were included in our analysis and reasons for exclusion were duplicate samples (*n* = 12) or unclassified tumours (*n* = 15, ER−/PR+/HER2− tumours). METABRIC clinical and genomic data are publicly available from the EGA-archive (https://ega-archive.org) under study number EGAS00000000083.

**Fig. 1:**
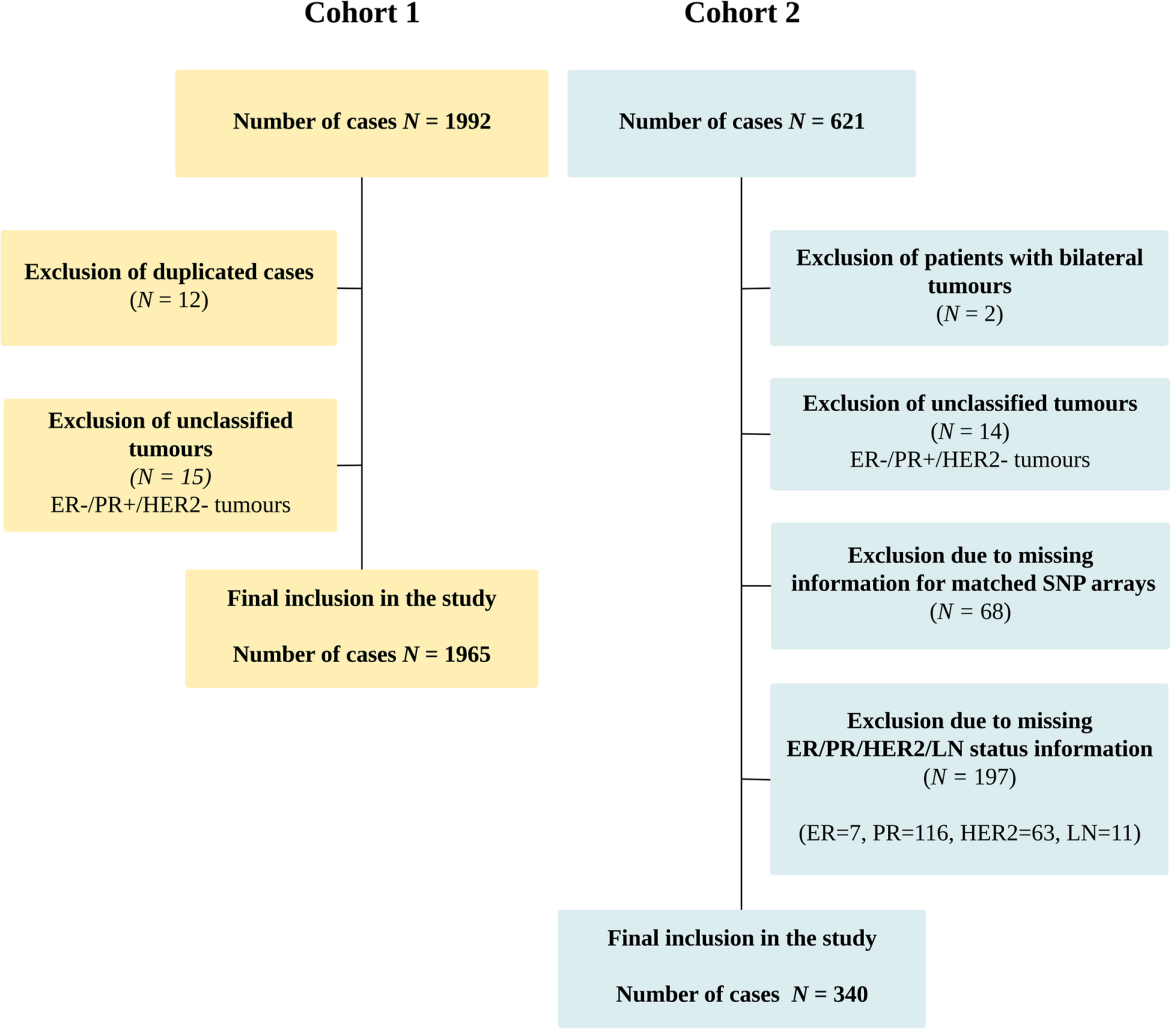
CONSORT diagram of patient selection in cohort 1 and cohort 2. ER oestrogen receptor, PR progesterone receptor, HER2 human epidermal growth factor receptor 2

Cohort 2 has also been previously extensively described [23]. Briefly, this cohort was derived from a nested case–control study and consists of 621 individuals diagnosed with primary breast tumours between January 1, 1997, and December 31, 2005. Of these, 340 were included in our analysis and reasons for exclusion were bilateral tumours (*n* = 2), unclassified tumours (*n* = 14, ER−/PR+/HER2− tumours), no matching SNP array (*n* = 68) and missing clinico-pathological data (*n* = 197). Median follow-up in this cohort is 14.4 years and is complete to January 10, 2015. Follow-up information was retrieved from the Stockholm–Gotland Breast Cancer Registry using a national registration number unique to all Swedish citizens. The clinical endpoint for both studies was breast cancer-specific survival (BCSS) defined as patients who have not died from breast cancer in the study period from the date of surgery to end of follow-up. Exclusion criteria for both cohorts are shown in the CONSORT diagram in Fig. 1.

### ER, PR and HER2

#### Cohort 1

Tumour ER and HER2 status was taken directly from the clinico-pathological data of the METABRIC study and is immunohistochemistry (IHC) based [25, 26]. PR status is based on a gene expression classification as described in the original publication [25].

#### Cohort 2

Primary breast tumour ER and PR status (assessed by IHC, > 10% cut-off for positivity) was collected from pathology reports. HER2 status was determined using chromogenic in situ hybridization (CISH) [27] on tissue microarrays (TMAs) and scored by a breast cancer pathologist. This study was performed and reported in accordance with the REporting recommendations for tumour MARKer prognostic studies (REMARK) guidelines [28], and biospecimen reporting for improved study quality (BRISQ) criteria for this cohort have been previously published [23].

### Genomic profiling

#### Cohort 1

Genomic profiling of METABRIC tumours was performed using whole genome gene expression (Illumina HT-12-v3 platform) and SNP arrays (Affymetrix SNP 6.0 platform) as detailed in the original publication [25].

#### Cohort 2

##### RNA gene expression profiling

The Qiagen RNeasy Mini Kit (Qiagen, Germany) was used for RNA extraction from frozen tumours, and samples were subsequently profiled for whole genome gene expression through hybridization to the HRSTA-2.0 custom human Affymetrix array GPL10379; see further details here [23].

##### DNA genotyping

Extraction was carried out using the QIAamp DNA mini kit (Qiagen, Germany) on frozen tumours, and genotyping was performed using the Human1M-Duo BeadChip (Illumina, CA, USA).

Both gene expression and genotyping studies in cohort 2 were approved by the ethics committee at Karolinska Institutet (Stockholm, Sweden).

### *CCND1* gene copy number analysis

#### Cohort 1

Normalised copy number variation (CNV) data and segmentation files (derived using circular binary segmentation—CBS) for METABRIC were downloaded from the EGA-archive.

#### Cohort 2

CNV data was generated from SNP array files using the CNVpartition (version 3.2.0) plugin from within GenomeStudio software (version 2011.1, Illumina, CA, USA). Similar to cohort 1, segmentation was performed using CBS as part of the R *DNAcopy* package [29]. Copy number alteration (CNA) calls for both cohorts were then derived by importing CNV, segmentation (derived from CBS) and platform-specific marker files to the Genomic Identification of Significant Targets in Cancer 2.0 (GISTIC) module [30]. GISTIC CNA amplification/deletion thresholds were set to ± 0.3, and regions with a *q* value of < 0.25 were considered significant. Of note, both of these cut-off values are in line with those used by The Cancer Genome Atlas for breast tumours [31]. *CCND1* amplification/deletion calls for each tumour in both cohorts were taken from the gene-level output files of the GISTIC algorithm and matched to clinico-pathological data.

### Genomic classifiers and differential gene expression

#### Cohort 1

PAM50 molecular subtype calls for METABRIC were taken from the original publication. Normalised microarray gene expression data for the METABRIC cohort was downloaded from the EGA-archive and subsequently used for differential gene expression (DGE) analysis. DGE was assessed between *CCND1*-amplified and non-amplified luminal A and luminal B tumours using the R package *Limma* [32], and only genes with an adjusted *P* value < 0.05 were considered significant.

#### Cohort 2

Microarray data was pre-processed and normalised using the R *aroma.affymetrix* package [33]; see further details here [23, 34]. Data has been deposited at the NCBI Gene Expression Omnibus under the accession number GSE48091. PAM50 was applied as described in the original publication [35].

### Statistical analysis

All statistical analyses were performed using R statistical software version 3.4.3 [36]. To assess differences between clinico-pathological variables and *CCND1* amplified/non-amplified tumours statistical tests were chosen based on the variable class being compared: nominal versus nominal—χ^**2**^; ordinal versus nominal—Mann–Whitney. Student’s *t* test was used to test for differences in mean gene expression between *CCND1-*amplified vs. non-amplified tumours within PAM50 subtypes. Similarly, ANOVA with post hoc Tukey was used for determining differences in mean gene expression across PAM50 subtypes. All tests were two-sided, and a *P* value of < 0.05 was considered statistically significant. Kaplan–Meier analysis was performed for *CCND1-*amplified and non-amplified tumours with 15-year breast cancer-specific survival as the clinical endpoint. Similarly, Cox multivariable proportional hazard analyses were used to determine survival differences between *CCND1-*amplified and non-amplified tumours with the latter as reference group and the same clinical endpoint. Every multivariable analysis was adjusted for tumour size, tumour grade, nodal status, endocrine treatment, PAM50 subtype and patient cohort.

## Results

### Clinico-pathological characteristics of *CCND1*-amplified tumours

In keeping with our aim to comprehensively determine the long-term prognostic and treatment predictive capacity of *CCND1* amplification, we analysed two cohorts of 1965 and 340 breast cancer patients respectively, with matching gene expression, SNP arrays and long-term (15 years) clinical follow-up. A CONSORT diagram of exclusion criteria for both cohorts is shown in Fig. 1, and clinico-pathological characteristics for both cohort split by *CCND1* amplification status are shown in Table 1.

**Table 1:** Clinico-pathological characteristics of patients in cohort 1 and 2 split by *CCND1* amplification status

Variable	Cohort 1 (*n* = 1965)		*P*	Cohort 2 (*n* = 340)		*P*
	Non-Amp	Amp		Non-Amp	Amp	
	*n* (%)	*n* (%)		*n* (%)	*n* (%)	
	1539 (78)	426 (22)		221 (65)	119 (35)	
ER						
Positive	1129 (73)	377 (88)	*< 0.001*	158 (71)	93 (78)	0.182
Negative	410 (27)	49 (12)		63 (29)	26 (22)	
PR						
Positive	794 (52)	231 (54)	0.364	140 (63)	71 (60)	0.504
Negative	745 (48)	195 (46)		81 (37)	48 (40)	
HER2						
Positive	190 (12)	57 (13)	0.626	40 (18)	30 (25)	0.122
Negative	1349 (88)	369 (87)		181 (82)	89 (75)	
Elston–Ellis grade						
I	148 (10)	22 (5)	0.200*	22 (10)	9 (8)	0.400*
II	625 (43)	142 (34)		102 (47)	47 (40)	
III	690 (47)	252 (61)		93 (43)	62 (52)	
Missing cases = 86				Missing cases = 5		
LN status						
Positive	748 (49)	188 (44)	0.114	116 (52)	81 (68)	*0.005*
Negative	791 (51)	238 (56)		105 (48)	38 (32)	
Tumour size						
< 20 mm	497 (33)	120 (28)	0.108	101 (47)	51 (43)	0.492
≥ 20 mm	1026 (67)	303 (72)		115 (53)	68 (57)	
Missing cases = 19				Missing cases = 5		
Age						
≤ 45	208 (14)	41 (10)	*0.006*	11 (12)	7 (10)	0.825
45–55	312 (20)	69 (16)		78 (83)	53 (82)	
≥ 55	1019 (66)	316 (74)		5 (5)	5 (8)	
				Missing cases = 181		
IHC subgroups						
ER+/LN−/HER2−	581 (38)	190 (45)	*< 0.001*	64 (29)	26 (23)	0.142
ER+/LN+/HER2−	482 (31)	145 (34)		75 (34)	47 (39)	
HER2+	190 (12)	57 (13)		40 (18)	30 (25)	
TN (ER−/PR−/HER2−)	286 (19)	34 (8)		42 (19)	16 (13)	
PAM50						
Luminal A	602 (39)	116 (27)	*< 0.001*	77 (35)	38 (32)	0.051
Luminal B	291 (19)	197 (46)		36 (16)	32 (27)	
HER2-enriched	184 (12)	54 (14)		30 (14)	20 (17)	
Basal-like	289 (19)	31 (7)		55 (25)	24 (20)	
Normal-like	168 (11)	27 (6)		23 (10)	5 (4)	
Missing cases = 6						
Treatments						
Endocrine therapy	773 (50)	249 (58)	*< 0.001*	59 (27)	33 (27)	0.950
Chemotherapy	196 (13)	25 (6)		60 (27)	30 (25)	
Both	148 (10)	41 (10)		99 (45)	55 (47)	
None	422 (27)	111 (26)		3 (1)	1 (1)	

Correlations were calculated using *X*^*2*^ test unless otherwise specified

*ER* oestrogen receptor alpha, *PR* progesterone receptor, *HER2* human epidermal growth factor 2 receptor, *TN* triple negative (ER−/PR−/HER2−), *LN* lymph node status, *Amp/non-Amp CCND1* amplified/non-amplified, *Both* patients sequentially received chemotherapy and endocrine therapy

* = Wilcoxon/Mann–Whitney

Twenty-two percent (426/1965, 22%) and 35% (119/340, 35%) of tumours in cohorts 1 and 2, respectively, were found to harbour *CCND1* amplifications, in line with previously published figures [7–11] (Table 1). Of note, as cohort 2 is enriched for patients with aggressive metastatic tumours, a higher percentage of *CCND1* amplifications was anticipated. In general, *CCND1-*amplified tumours were more likely to be ER-positive and of luminal subtype relative to non-amplified tumours (Table 1). It is, however, worth noting that amplified tumours were also present in ER-negative and non-luminal breast cancer subtypes.

### *CCND1* amplification predicts poor long-term survival in ER+ breast cancer patient subgroups (cohort 1)

**Fig. 2:**
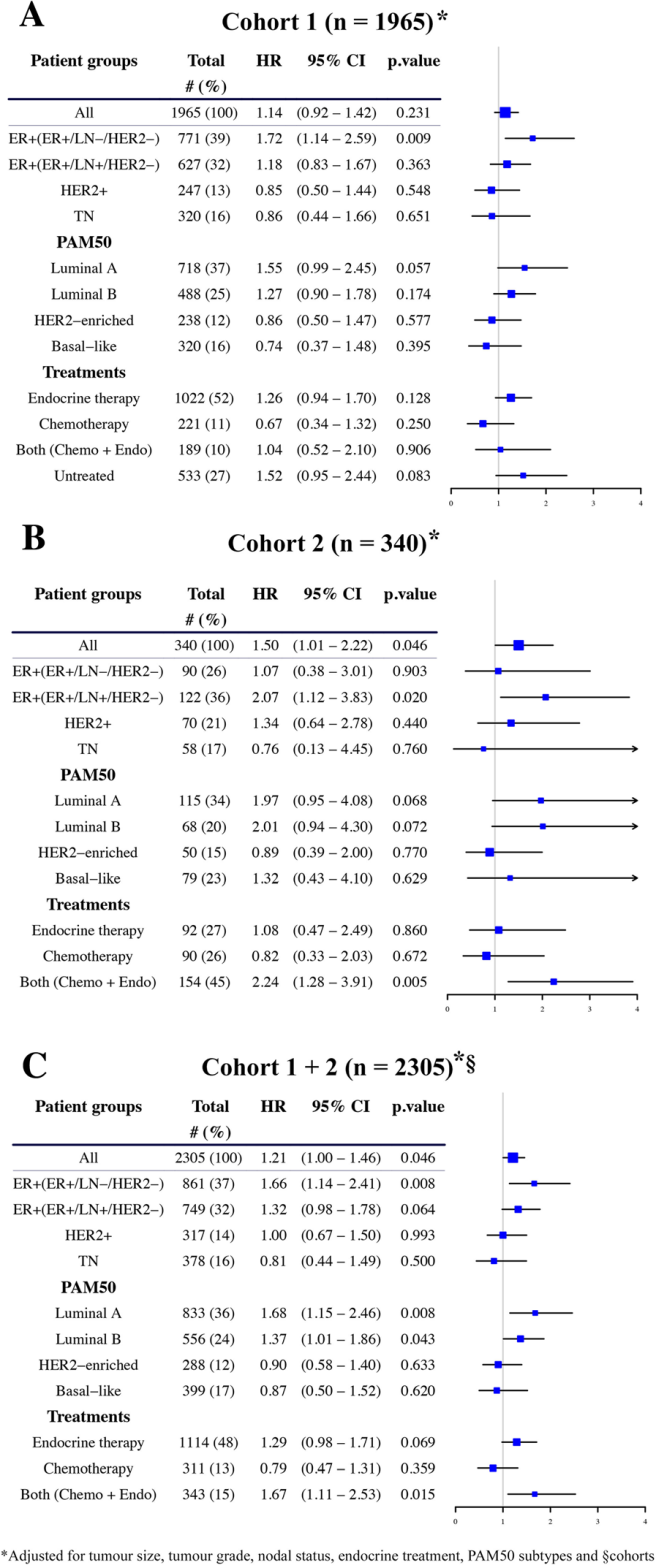
Forest plots of log hazard ratios (HR) for breast cancer-specific survival. Multivariate Cox proportional hazards regression models in **a** cohort 1, **b** cohort 2 and **c** both cohorts combined. ER+/− oestrogen receptor positive/negative, HER2+/− human epidermal growth factor positive/negative, PR progesterone receptor, TN triple negative (ER−/PR−/HER2−), LN+/− lymph node metastasis positive/negative

Next, in cohort 1, we examined the relationship between *CCND1* amplification and long-term BCSS in clinically relevant patient subgroups defined by IHC/nodal status (all, ER+/LN−/HER2, ER+/LN+/HER2−, HER2+ or TNBCs), PAM50 gene expression subtype (luminal A/B, HER2-enriched or basal-like) or treatment received (endocrine therapy, chemotherapy, both sequentially or untreated). In Kaplan–Meier analysis *CCND1*-amplifed patients were found to have a worse 15-year BCSS in the IHC/nodal subgroups ER+/LN−/HER2− and ER+/LN+/HER2− (Additional file 1: Figure S1B and C, *P* <  0.001 and *P* = 0.016, respectively). Similarly, *CCND1* amplification was associated with poorer survival in luminal A—with a notable deviation between survival curves after 5 years, endocrine-treated and untreated breast cancer patients (Additional file 2: Figure S2A, E, and H, *P* = 0.019, 0.007 and 0.014, respectively). In multivariable Cox regression analysis, this statistical significance remained for ER+/LN−/HER2− (HR = 1.72, 95% CI, 1.14–2.59, Fig. 2a) patients only; however, trends were observed for luminal A (HR = 1.55, 95% CI, 0.99–2.45) and untreated subgroups (HR = 1.52, 95% CI, 0.95–2.44).

### Similar results in a second smaller cohort (cohort 2) and when combining both cohorts (cohorts 1 + 2)

Similar trends were found for *CCND1*-amplified tumours in Kaplan–Meier (Additional file 3: Figure S3 and Additional file 4: Figure S4) and multivariable (Fig. 2b) analyses of a second independent cohort of 340 patients; however, smaller patient numbers resulted in wider confidence intervals and reduced statistical power. In multivariable analysis, patients with ER+/LN+/HER2− *CCND1*-amplified tumours were found to have worse 15-year BCSS (HR = 2.07, 95% CI, 1.12–3.83), and comparable trends were noted in luminal A (HR = 1.97, 95% CI, 0.95–4.08) and luminal B (HR = 2.01, 95% CI, 0.94–4.30) subgroups. *CCND1*-amplified ER+/LN−/HER2− patients did not show poorer survival in this cohort (HR = 1.07, 95% CI, 0.38–3.01) (Fig. 2b); however, the size of this subgroup is considerably less (*n* = 90) than cohort 1 (*n* = 771).

Combining both cohorts to increase patient numbers demonstrated poorer BCSS for *CCND1*-amplified ER+/LN−/HER2− (HR = 1.66, 95% CI, 1.14–2.41, Fig. 2c), luminal A (HR = 1.68, 95% CI, 1.15–2.46) and luminal B (HR = 1.37, 95% CI, 1.01–1.86) subgroups, along with trends for ER+/LN+/HER2− (HR = 1.32, 95% CI, 0.98–1.78) and endocrine-treated patients (HR = 1.29, 95% CI, 0.98–1.71). These results highlight the ability of *CCND1* amplification status to select a group of patients with poor 15-year breast cancer-specific survival. Of note, results were more ambiguous for systemically treated *CCND1*-amplified patients (see group “Both (Chemo + Endo)”) as no difference in survival was noted in cohort 1 (HR = 1.04, 95% CI, 0.52–2.10, Fig. 2a) and significantly worse survival was found in cohort 2 and combined cohorts (HR = 2.24, 95% CI, 1.28–3.91, Fig. 2b and HR = 1.67, 95% CI, 1.11–2.53, Fig. 2c, respectively).

### *CCND1*-amplified luminal A tumours display gene expression changes consistent with more aggressive tumours

**Fig. 3:**
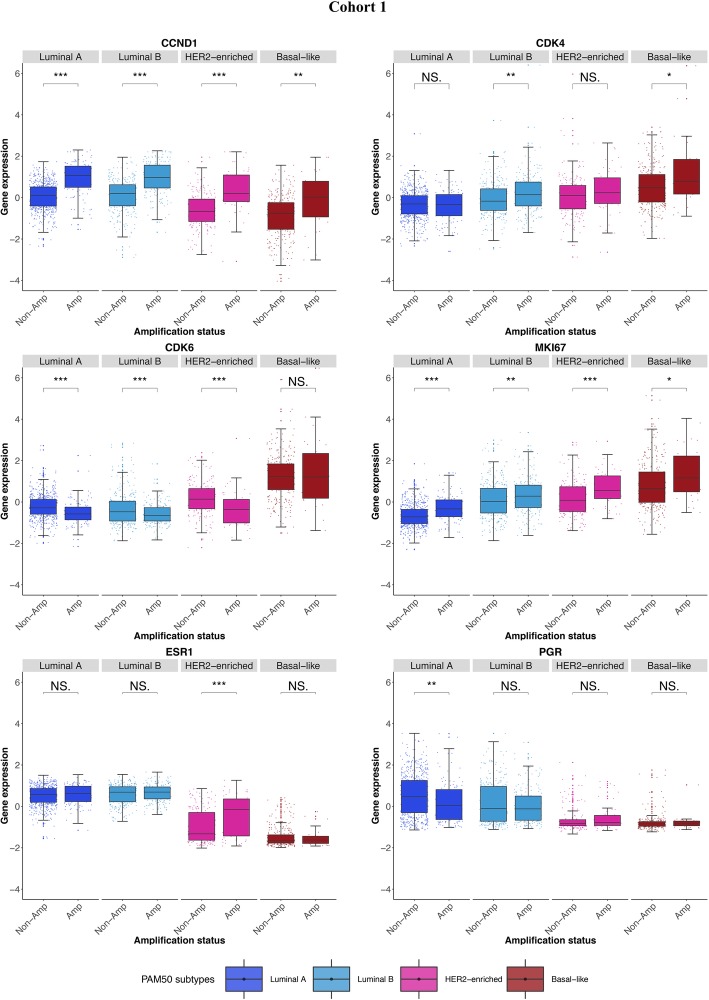
Boxplots comparing the expression of *CCND1*, *CDK4/6*, *MKI67*, *ESR1* and *PGR* within PAM50 subtypes. CCND1 cyclin D1 gene, CDK4/6 cyclin-dependent kinase 4/6 genes, MKI67 marker of proliferation KI-67 protein coding gene, ESR1 oestrogen receptor 1 gene, PGR progesterone receptor gene, Amp/Non-Amp CCND1 amplified/non-amplified tumours, *P* values (based on two-sided Student’s *t* test) = NS > 0.05, * < 0.05, ** < 0.01, *** < 0.001

In order to understand why *CCND1* amplification confers a worse survival in luminal A tumours, we first examined the expression of genes related to the cell cycle and cell proliferation across all tumours of cohort 1 within the context of the PAM50 subtypes. *CCND1* gene expression was highest in luminal A/B and lowest in basal-like tumours, while the opposite was true for *CDK4/6* and the proliferation marker gene *MKI67* (Additional file 5: Figure S5). Similarly to *CCND1*, expression of the cell cycle-related genes *RB1* (retinoblastoma 1) and *AR* (androgen receptor) were also higher in luminal tumours relative to basal-like (Additional file 5: Figure S5, see “AR” and “RB1”, *P* <  0.001 for luminal A vs. basal-like comparisons). Taken together, these findings suggest that the high levels of proliferation seen in basal-like tumours are unlikely driven by traditional *CCND1*/*RB1* signalling. As expected, expression of the oestrogen (*ESR1*)/progesterone (*PGR*) and HER2 (*ERBB2*) genes were highest in the luminal and HER2-enriched subtypes respectively, and as shown by others [37], expression of *PGR* was lower in luminal B tumours relative to luminal A (Additional file 5: Figure S5, “PGR”, *P* <  0.001). Examining the same genes in *CCND1*-amplified vs. non-amplified luminal A tumours showed increased *MKI67* and decreased *PGR* gene expression in amplified tumours (Fig. 3, see “MKI67” and “PGR”, *P* <  0.001 and *P* = 0.002 respectively), consistent with a more aggressive phenotype. This may partially explain why luminal A *CCND1*-amplified tumours demonstrate poorer survival relative to their non-amplified counterparts. Interestingly, *CDK4* gene expression was higher in luminal B and basal-like *CCND1*-amplified tumours only (Fig. 3, “CDK4” *P* = 0.004 and *P* = 0.029 respectively) whilst *CDK6* expression was lower in luminal A, luminal B and HER2-enriched *CCND1*-amplified tumours (Fig. 3, “CDK6” *P* <  0.001 for all comparisons). No differences were found between *CCND1*-amplified vs. non-amplified tumours in any subtype for the *AR* or *RB1* genes (Additional file 6: Figure S6).

Finally, we further characterised luminal A *CCND1*-amplified tumours by determining if any shared genes exist between amplified tumours in luminal A vs. luminal B molecular subgroups. Differential gene expression analyses were performed between *CCND1*-amplified vs. non-amplified tumours in luminal A and B tumours separately, and the top most changed genes are shown in Additional file 7: Table S1. Remarkably, the top three differentially expressed genes are identical in both luminal A and B subtypes and 12 of the top 20 genes also overlap and are consistent with an 11q amplification event (Additional file 7: Table S1). These results highlight the similarities between luminal A and B *CCND1*-amplified tumours.

## Discussion

In this study, we integrated DNA copy number data from SNP assays, RNA expression data from whole genome transcriptome arrays and long-term survival data from over 2305 breast cancer patients, with the central goal of determining the prognostic and treatment predictive capacity of cyclin D1 gene amplification. In what is, to our knowledge, the largest and most comprehensive analysis of *CCND1* amplification to date, two main novel findings were observed. First, ER+/LN−/HER2−, luminal A and B breast cancer patients with a *CCND1*-amplified tumour show worse 15-year BCSS relative to non-amplified patients. Similar statistical trends were observed for ER+/LN+/HER2− and endocrine-treated patients. Second, luminal A *CCND1*-amplified tumours display gene expression changes consistent with more aggressive tumours, specifically increased *MKI67* and decreased *PGR* gene expression in addition to an overlap in genes differentially expressed in *CCND1*-amplified luminal B tumours.

These findings are supported by the work of others who have also shown poor survival for patients with ER-positive *CCND1*-amplified tumours [8–10, 12, 15], albeit with shorter clinical follow-up relative to our cohorts. In particular, results from another large study—TransATAC (*n* = 1155)—are in line with our conclusions and show an increased risk of recurrence at 10 years in ER-positive endocrine-treated patients with *CCND1*-amplified tumours [9]. Focusing on luminal A tumours, Holm et al. also noted worse 10-year overall survival for *CCND1*-amplified luminal A tumours in univariate analysis [13] (*n* = 12 *CCND1*-amplified and 78 non-amplified) as did Chin et al. when examining the amplification status of the 11q13 *CCND1* amplicon with a median overall survival follow-up of 6.6 years [38]. Patient numbers in both of these studies were however too low to perform multivariable analyses.

It has been previously been suggested that *CCND1* amplification could serve as a biomarker for the prediction of response to cyclin-dependent kinase 4/6 inhibitors [39]. When comparing *CCND1*-amplified vs. non-amplified tumours, we found that *CCND1*-amplified tumours show higher expression of the proliferation gene *MKI67* across all PAM50 subtypes, higher *CDK4* expression in luminal B and basal-like subtypes only and lower *CDK6* expression in luminal A, luminal B and HER2-enriched subtypes. These findings imply that the increase in proliferation found in *CCND1*-amplified tumours is unlikely to be dependent on the upregulation of *CDK4/6*. As such, our data indicate that *CCND1* amplification may perform poorly as a predictive biomarker in this setting. This hypothesis is supported by patient data from the PAMOLA-1 study showing no benefit of palbociclib in patients whose tumours were *CCND1*-amplified [40].

There are a number of strengths to our analyses; first, as this is the largest study of its kind, the number of patients in clinically relevant and PAM50 subtypes has allowed us to comprehensively characterise the prognostic and predictive potential of *CCND1* amplification using multivariable adjusted statistics; second, multivariable results are generally analogous across two independent breast cancer cohorts with a 15-year BCSS endpoint; third, matching gene expression and SNP array data has meant that we have been able to provide biological insight as to why *CCND1*-amplified tumours may confer worse survival; and fourth, we have kept methods for amplification calls (using CBS, GISTIC) as similar as possible between both cohorts in order to ensure the reproducibility and consistency of our findings. The limitations are as follows: our analyses are retrospective in nature, we have not performed adjustment for multiple testing and our second cohort is substantially smaller than our first and enriched for more aggressive tumours, ultimately resulting in wider confidence intervals and statistical trends rather than formal significance for some subgroups.

## Conclusions

In summary, we show that assessment of *CCND1* amplification status can provide long-term independent prognostic information in patients with ER+/LN−/HER2− tumours, and novelly, within luminal A and luminal B tumours. These findings highlight the potential of *CCND1* to pinpoint patients with poor long-term survival that could benefit from more aggressive clinical treatment strategies.

## Additional files

Additional file 1: **Figure S1.** Survival analysis (Kaplan–Meier estimates) with breast cancer-specific survival (BCSS) as a clinical end point for all patients of cohort 1 split by IHC subtypes. (A) All patients, (B) ER+/LN−/HER2−, (C) ER+/LN+/HER2−, (D) HER2+, and (E) TN. ER oestrogen receptor alpha, PR progestrone receptor, HER2 human epidermal growth factor receptor 2, LN lymph node metastasis, TN triple negative (ER−/PR−/HER2−). *P* values refer to log-rank tests. (TIF 3090 kb)

Additional file 2: **Figure S2.** Survival analysis (Kaplan–Meier estimates) with breast cancer-specific survival (BCSS) as a clinical end point for all patients of cohort 1 split by PAM50 subtypes and treatments. (A) Luminal A, (B) luminal B, (C) HER2-enriched, (D) basal-like, (E) endocrine therapy, (F) chemotherapy, (G) both chemotherapy and endocrine combined, (H) untreated patients. *P* values refer to log-rank tests. (TIF 3311 kb)

Additional file 3: **Figure S3.** Survival analysis (Kaplan–Meier estimates) with breast cancer-specific survival (BCSS) as a clinical end point for all patients of cohort 2 split by IHC subtypes. (A) All patients, (B) ER+/LN−/HER2−, (C) ER+/LN+/HER2−, (D) HER2+, (E) TN. ER oestrogen receptor alpha, PR progestrone receptor, HER2 human epidermal growth factor receptor 2, LN lymph node metastasis, TN triple negative (ER−/PR−/HER2−). *P* values refer to log-rank tests. (TIF 2991 kb)

Additional file 4: **Figure S4.** Survival analysis (Kaplan–Meier estimates) with breast cancer-specific survival (BCSS) as a clinical end point for all patients of cohort 2 split by PAM50 subtypes and treatments. (A) Luminal A, (B) luminal B, (C) HER2-enriched, (D) basal-like, (E) endocrine therapy, (F) chemotherapy, (G) both chemotherapy and endocrine combined, (H) untreated patients. *P* values refer to log-rank tests. (TIF 3194 kb)

Additional file 5: **Figure S5.** Box plots comparing the expression of *CCND1*, *CDK4/6*, *MKI67*, *RB1*, *AR*, *ESR1*, *PGR* and *ERBB2* genes split by PAM50 subtypes in cohort 1. CCND1 cyclin D1 gene, CDK4/6 cyclin-dependent kinase 4/6 genes, MKI67 marker of proliferation KI-67 protein-coding gene, RB1 retinoblastoma 1 gene, AR androgen receptor gene, ESR1 oestrogen receptor 1 gene, PGR progestrone receptor gene, ERBB2 human epidermal growth factor receptor 2 gene, *P* values (based on ANOVA with post hoc Tukey HSD test) = NS > 0.05, * <  0.05, ** <  0.01, *** < 0.001 (JPG 4941 kb)

Additional file 6: **Figure S6.** Box plots comparing the expression of *ERBB2*, *AR* and *RB1* genes split by PAM50 subtypes in CCND1-amplified/non-amplified tumours in cohort 1. CCND1 cyclin D1 gene, ERBB2 human epidermal growth factor receptor 2 gene, AR androgen receptor gene, RB1 retinoblastoma 1 gene, *P* values (based on two-sided Student’s *t* test) = NS > 0.05, * < 0.05, ** < 0.01, *** < 0.001 (JPG 2924 kb)

Additional file 7: **Table S1.** Common differentially expressed genes among the top 20 hits of cohort 1 (luminal A, luminal B). Common genes are shown in bold; Chr.loc, chromosomal location; FDR Adj *P*, the false discovery rate adjusted *P* values derived from Benjamini–Hochberg correction; logFC, logarithmic fold change; *Complementary information derived from The HUGO Gene Nomenclature Committee (HGNC) and Gene-Card databases. (PDF 66 kb)

## Abbreviations

AR: Androgen receptor
BCSS: Breast cancer-specific survival
BRISQ: Biospecimen reporting for improved study quality
CBS: Circular binary segmentation
CDK4/6: Cyclin-dependent kinases 4/6
CISH: Chromogenic in situ hybridization
CNA: Copy number alteration
CNV: Copy number variation
DGE: Differential gene expression analysis
ER: Oestrogen receptor alpha
ESR1: Oestrogen receptor 1
GISTIC: Genomic Identification of Significant Targets in Cancer
HER2: Human epidermal growth factor 2
HR: Hazard ratio
IHC: Immunohistochemistry
LN: Lymph node
MKI67: Marker Of Proliferation Ki-67
PGR: Progesterone
RB1: Retinoblastoma 1
REMARK: REporting recommendations for tumour MARKer prognostic studies
TMA: Tissue microarray
TNBC: Triple-negative breast cancer
