# Supplementary material for: The long-term prognostic and predictive capacity of cyclin D1 gene amplification in 2305 breast tumours
Source: Breast Cancer Res. 2019 Feb 28;21:34. doi: 10.1186/s13058-019-1121-4 (PMC6394106; doi:10.1186/s13058-019-1121-4)
Supplement: Supplementary file 7 — Table S1. Common differentially expressed genes among the top 20 hits of cohort 1 (luminal A, luminal B). Common genes are shown in bold; Chr.loc, chromosomal location; FDR Adj P, the false discovery rate adjusted P values derived from Benjamini–Hochberg correction; logFC, logarithmic fold change; *Complementary information derived from The HUGO Gene Nomenclature Committee (HGNC) and Gene-Card databases. (PDF 66 kb) [file 13058_2019_1121_MOESM7_ESM.pdf]

Table S1.

Common differentially expressed genes among top 20 hits Cohort 1 (Luminal A, Luminal B)

Luminal A						Luminal B					
**Entrez-ID**	**Gene-symbol**	**logFC**	*P*	**FDR-Adj** *P*	**Chr.loc**	**Entrez-ID**	**Gene-symbol**	**logFC**	*P*	**FDR-Adj** *P*	**Chr.loc**
8500	**PPFIA1**	-1.0349	< 0.001	< 0.001	11 q13.3	8500	**PPFIA1**	-1.0908	< 0.001	< 0.001	11 q13.3
220064	**ORAOV1**	-1.0455	< 0.001	< 0.001	11 q13.3	220064	**ORAOV1**	-1.1347	< 0.001	< 0.001	11 q13.3
2017	**CTTN**	-0.9051	< 0.001	< 0.001	11 q13.3	2017	**CTTN**	-0.9812	< 0.001	< 0.001	11 q13.3
219927	**MRPL21**	-0.6913	< 0.001	< 0.001	11 q13.3	595	**CCND1**	-0.9660	< 0.001	< 0.001	11 q13.3
219931	**TPCN2**	-0.4768	< 0.001	< 0.001	11 q13.3	55107	ANO1	-0.7996	< 0.001	< 0.001	11 q13.3
595	**CCND1**	-0.9865	< 0.001	< 0.001	11 q13.3	219927	**MRPL21**	-0.5501	< 0.001	< 0.001	11 q13.3
5499	**PPP1CA**	-0.3623	< 0.001	< 0.001	11 q13.2	84881	**RPUSD4**	0.3211	< 0.001	< 0.001	11 q24.2
5883	**RAD9A**	-0.5090	< 0.001	< 0.001	11 q13.2	219931	**TPCN2**	-0.4087	< 0.001	< 0.001	11 q13.3
219854	**TMEM218**	0.3954	< 0.001	< 0.001	11 q24.2	5499	**PPP1CA**	-0.3326	< 0.001	< 0.001	11 q13.2
3508	**IGHMBP2**	-0.2556	< 0.001	< 0.001	11 q13.3	29087	THYN1	0.3237	< 0.001	< 0.001	11 q25.0
84881	**RPUSD4**	0.3709	< 0.001	< 0.001	11 q24.2	5883	**RAD9A**	-0.4282	< 0.001	< 0.001	11 q13.2
55291	PPP6R3	-0.4355	< 0.001	< 0.001	11 q13.2	27034	ACAD8	0.2958	< 0.001	< 0.001	11 q25.0
9986	**RCE1**	-0.3334	< 0.001	< 0.001	11 q13.2	219854	**TMEM218**	0.3089	< 0.001	< 0.001	11 q24.2
51111	SUV420H1	-0.4000	< 0.001	< 0.001	11 q13.2	3508	**IGHMBP2**	-0.2208	< 0.001	< 0.001	11 q13.3
1798	DPAGT1	0.3832	< 0.001	< 0.001	11 q23.3	112936	VPS26B	0.2919	< 0.001	< 0.001	11 q25.0
9633	MTL5	-0.3964	< 0.001	< 0.001	11 q13.3	6768	ST14	0.3724	< 0.001	< 0.001	11 q24.3
1374	CPT1A	-0.3565	< 0.001	< 0.001	11 q13.3	9986	**RCE1**	-0.3577	< 0.001	< 0.001	11 q13.2
83480	PUS3	0.3108	< 0.001	< 0.001	11 q24.2	9088	PKMYT1	-0.2561	< 0.001	< 0.001	16 p13.3
92105	INTS4	-0.4786	< 0.001	< 0.001	11 q14.1	143879	KBTBD3	0.2024	< 0.001	< 0.001	11 q22.3
28971	AAMDC	-0.6462	< 0.001	< 0.001	11 q14.1	54970	TTC12	0.4104	< 0.001	< 0.001	11 q23.2

Common genes are shown in bold; Chr.loc = Chromosomal location; FDR Adj *P* = The false discovery rate adjusted *P* values dervied from Benjamini-Hochberg correction; logFC = logarithmic fold-change

* Complementary information derived from The HUGO Gene Nomenecleture Committee (HGNC) and Gene-Card databases
